# Construction of a predictive model for rebleeding risk in upper gastrointestinal bleeding patients based on clinical indicators such as *Helicobacter pylori* infection

**DOI:** 10.3389/fmicb.2025.1510126

**Published:** 2025-05-14

**Authors:** Wei Zang, Ze Lin, Yanduo Zhao, Tianshi Jia, Xinglong Zhang

**Affiliations:** ^1^Department of Radiology, ShengJing Hospital of China Medical University, Shenyang, Liaoning, China; ^2^The Second Clinical College of China Medical University, Shenyang, Liaoning, China; ^3^The Fourth Affiliated Hospital of China Medical University, Shenyang, Liaoning, China

**Keywords:** deep learning, UGIB, *Helicobacter pylori*, Rockall score, Glasgow-Blatchford score, AIMS65 score

## Abstract

**Background:**

The annual incidence of upper gastrointestinal hemorrhage (UGIB) is about 60 cases/100,000 people, and about 40% of UGIB patients have hemorrhagic ulcers. Ulcer formation is often associated with *Helicobacter pylori* (*H. pylori*) infection, non-steroidal anti-inflammatory drugs (NSAIDs) use and other factors, so ulcerative disease is the main cause of upper gastrointestinal bleeding. *H. pylori* induces chronic superficial gastritis with neutrophils infiltrating into the mucosa, so it is assumed that *H. pylori* infection is the basis of bleeding lesions. *H. pylori* infection is widespread worldwide, with about 50% of the population carrying the bacteria. Mortality during hospitalization is higher in patients with UGIB because rebleeding significantly increases the risk of death, especially if timely intervention is not provided. Rebleeding may also lead to severe complications such as shock and multiple organ failure. At present, the commonly used clinical scores for UGIB patients mainly include Rockall score (RS), AIMS65 score and Glasgow-Blatchford score (GBS). Because some hospitals are limited by local medical and health conditions, they lack timely and accurate endoscopic diagnosis and treatment equipment, and it is difficult to make accurate and timely judgments on patients.

**Method:**

In this experiment, 254 patients with upper digestive tract hemorrhage from Shengjing Hospital affiliated to China Medical University were collected, and the clinical indicators and information of *H. pylori* infection, age, shock state, concomitant disease, *H. pylori* infection degree, systolic blood pressure, blood urea nitrogen, hemoglobin, pulse, black stool, syncope, liver disease and other patients were finally collected. We analyzed the correlation between various clinical indicators and rebleeding in hospitalized patients. Based on the collected clinical information and laboratory indicators, this study constructed a deep learning model, the data is divided into four categories (clinical information, vital signs, laboratory examination items, stool examination) as input, and Transformer is used as feature extractor. KAN as a classifier to predict the risk of rebleeding in patients with upper gastrointestinal bleeding. The model uses five-fold cross validation and calculates key metrics such as accuracy to evaluate its performance. In addition, the deep learning model was compared with a variety of machine learning methods (decision tree, random forest, logistic regression, K-nearest neighbor) and common clinical risk scores (Rockall score, AIMS65 score, Glasgow-Blatchford score) to verify its effectiveness and advantages. In order to highlight the importance of *H. pylori* infection degree to the model performance, we conducted a comparative experiment to observe the role of *H. pylori* infection degree in the model.

**Results:**

In the correlation analysis between rebleeding and clinical data and related indicators, the risk of rebleeding in men (62.5%) was higher than that in women (43.47%), and the risk of rebleeding in patients with concurrent diseases (60.37%) was higher than that in patients without concurrent diseases. In the analysis of the correlation between the degree of infection and the laboratory test items, the hemoglobin level of patients will also change with the change of the degree of infection of patients (*p* < 0.05 in the above correlation analysis, all had statistical significance). The rebleeding detection rates of Rockall score, AIMS65 score and Glasgow Blatchford score were 16.14%, 0 and 77.17%, respectively. Of the four machine learning models, Random Forest (RF) had the highest accuracy on the test set at 0.68. The accuracy of the deep learning model on the verification set is the highest of 0.9750, and the accuracy of the test set is the highest of 0.9615. In addition, by exploring the influence of infection on the model prediction, it was found that the prediction accuracy of rebleeding in the non-*H. pylori* infection group (0.8989) was lower than that in the *H. pylori* infection group (0.9636), and other evaluation parameters were also lower than that in the infection group. In addition, by adding irrelevant random noise to mask the influence of infection degree on model output, it is found that the model prediction accuracy (0.7992) is significantly reduced.

**Conclusion:**

Based on the degree of *H. pylori* infection in patients with upper gastrointestinal bleeding, combined with a number of clinical laboratory tests and clinical data, we developed a clinical model for predicting the risk of rebleeding in patients with upper gastrointestinal bleeding. It provides an early prediction of rebleeding during a patient’s hospitalization and optimizes early intervention for patients to a certain extent. It provides a more concise, convenient and effective guidance scheme for small and medium-sized hospitals to make clinical decisions for UGIB patients.

## 1 Introduction

Upper gastrointestinal hemorrhage (UGIB) refers to the occurrence of blood in the esophagus, stomach and duodenum, and the clinical symptoms of hematemesis or black stool (Lanas et al., 2009; Thiebaud et al., 2017). It is estimated that the annual incidence of UGIB is about 60 cases / 100,000 people (Gu et al., 2023; Laine et al., 2012), among which gastric and duodenal ulcers are the common causes of UGIB, according to statistics, about 30% to 50% of UGIB cases are related to peptic ulcers (Kamboj et al., 2019). Ulcer formation is often associated with *H. pylori* infection, the use of non-steroidal anti-inflammatory drugs (NSAIDs), and excessive alcohol consumption. Studies have shown that *H. pylori* can induce chronic superficial gastritis, and neutrophils infiltrate the mucosa (Popa et al., 2021). *H. pylori* infection will damage the inner wall of blood vessels, resulting in impaired vascular skin function, resulting in decreased vascular tension, and promoting the development of acute upper gastrointestinal bleeding. Moreover, under the action of *H. pylori*, a large number of inflammatory factors will be produced, resulting in increased inflammation and a large number of platelet aggregation in blood vessels. It can cause thrombosis, block blood vessels and affect blood clotting function (Toews et al., 2024). Therefore, it is speculated that *H. pylori* infection is the basis of bleeding lesions. According to current guidelines, patients with UGIB should undergo esophagogastroduodenoscopy (EGD) within 12 to 24 h after hemodynamic resuscitation (Gralnek et al., 2021; Karstensen et al., 2020). When erythema with recent bleeding is observed through EGD, endoscopic treatment is required to reduce mortality, recurrent bleeding, and surgical intervention rates (Veisman et al., 2022). Mortality and risk of rebleeding in UGIB patients should be evaluated after endoscopic and laboratory examinations. Mortality during hospitalization is higher in patients with UGIB because rebleeding significantly increases the risk of death, especially if timely intervention is not provided. Rebleeding may also lead to severe complications such as shock and multiple organ failure (Chen et al., 2021; Matsuhashi et al., 2021; Shung and Laine, 2024). Common clinical scores include Rockall score (RS), AIMS65 score, and Glasgow-Blatchford score (GBS). RS score is mainly used for risk assessment of rebleeding and death, and a number of clinical prediction models rely on RS score. The accuracy of this score has always been an advantage due to its endoscopy project (Redondo-Cerezo et al., 2020; Seo et al., 2020; Shung et al., 2020), but many hospitals are limited by their own conditions, and it is difficult to achieve timely and effective use of endoscopy. Therefore, GBS score and AIMS65 score independent of endoscopy project have wider application scenarios, high sensitivity, good generality, simple economy, suitable for early application in emergency treatment, and can also be used to predict UGIB inpatient mortality (Stanley et al., 2017), while GBS and AIMS65 score are not as accurate as RS score. This brings some difficulties to the diagnosis and treatment of patients (Alali et al., 2023; Cazacu et al., 2023). Deep learning is a kind of machine learning method based on artificial neural network, its core idea is to automatically extract and represent complex features of data through multi-layer neural network structure. Deep learning implements complex feature learning through invisible transformations between layers and deep learning models automatically extract useful features from inputs through multi-system neural networks, avoiding the strong dependence on feature engineering in traditional machine learning (Koetzier et al., 2023; LeCun et al., 2015; Schmidhuber, 2015). In recent years, the diagnosis and treatment of digestive system diseases has been fully developed through deep learning, which mostly focuses on the diagnosis and treatment of digestive system tumors and the establishment of deep learning models through endoscopic content, and the semi-automatic or fully automated diagnosis and treatment of diseases (Dong et al., 2022; Kim et al., 2023; Zhang et al., 2023; Zhuang et al., 2022).

However, the current deep learning models using clinical indicators are still not completely divorced from endoscopy, but there are few studies on *H. pylori* infection. Therefore, this study established a deep learning model by obtaining the *H. pylori* infection situation of patients with upper gastrointestinal bleeding and combining with a number of clinical indicators to assess the risk of rebleeding in patients with UGUB in the absence of endoscopic information.

## 2 Methods

### 2.1 Inclusion criteria

**Figure 1:**
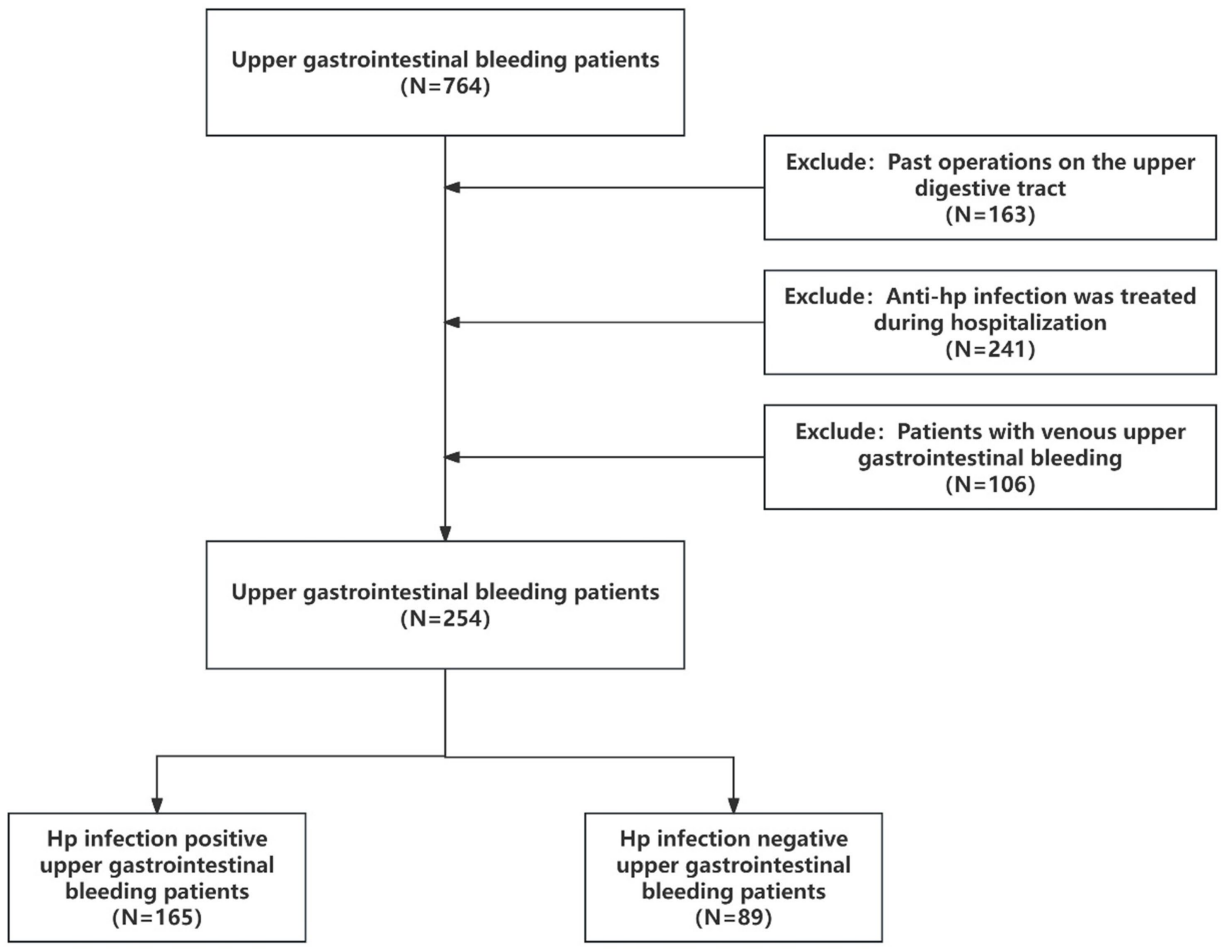
Flow chart of admission and discharge.

A total of 254 UGIB patients admitted to Shengjing Hospital Affiliated to China Medical University from January 2017 to June 2024 were selected and divided into *H. pylori* infection group and *H. pylori* non-infection group according to whether they were infected with *H. pylori*. There were 165 patients in *H. pylori* infection group and 89 patients in *H. pylori* non-infection group. The patients with *H. pylori* infection UGIB were further divided into weak positive (17 cases), positive (4 cases), weak positive for active infection (42 cases) and positive for active infection (102 cases). Inclusion criteria for UBIG patients: (1) Clinical manifestations included hematemesis, black stool, shock or other gastrointestinal bleeding symptoms; (2) Decreased hemoglobin level, decreased coagulation function, stool occult blood and other laboratory positive tests; (3) CT and endoscopy indicated upper gastrointestinal bleeding; (4) *H. pylori* infection was examined after hospitalization. Exclusion criteria: (1) Patients with past operations on upper gastrointestinal tract; (2) Anti-*H. pylori* infection treatment during hospitalization; (3) Patients with venous upper gastrointestinal bleeding. The flow chart of the row is shown in Figure 1. Detection of *H. pylori* infection in UGIB patients based on serological testing (antibody testing) and detection of *H. pylori* electrophoresis IgG antibodies. If the patient had *H. pylori* before and had anti-*H. pylori* infection, he was previously infected with *H. pylori*; if the patient had no *H. pylori* infection detected in the past and no anti-*H. pylori* infection in the past, he was currently infected with *H. pylori*. Combined with high titer warning and breath test, the infection was divided into positive and weak positive.

### 2.2 Rating

#### 2.2.1 Rockall rating

Rockall belongs to the post-endoscopic risk score, which was published in 1996 and was designed to predict mortality based on 4,185 UGIB cases. The full score ranges from 0 to 11 and includes five variables, two of which depend on endoscopy results: age, hemodynamic stability, comorbidities, endoscopic diagnosis, and erythema of recent bleeding (Rockall et al., 1996). According to the score, patients can be divided into high risk (≥5 scores), medium risk (3–4 scores), and low risk (0–2 scores). Specific scores are shown in Table 1.

**Table 1:** Content of Rockall rating.

Variables	Score
Age	
60–79 years old	1
>80 years old	2
Shock index	
Heart rate (beats per minute) > 100 and	1
Systolic blood pressure (mmHg) > 100	
Heart rate (beats per minute) > 100 and	2
Systolic blood pressure (mmHg) < 100	
Concomitant disease	
Heart failure, ischemic cardiomyopathy and other important concomitant diseases	2
Liver failure, kidney failure and disseminated malignancy	3
Endoscopic diagnosis	
Mallory Weiss had lacerations, no lesions	0
All other diagnoses	1
Malignant tumors of the upper digestive tract	2
Erythema of recent bleeding	
None, only dark spots	0
Blood, adhesion clot, ejection blood vessel	2

#### 2.2.2 Blatchford rating

GBS belongs to the preendoscopic risk score, which was published in 2000 and is based on 1,748 UGIB patients with a full score of 0 to 23 and includes eight variables: blood urea nitrogen; Hemoglobin; Systolic blood pressure; Heart rate; Black stool; Fainting; Presence of liver disease (known history or clinical/laboratory evidence) and heart disease (known history or clinical/laboratory evidence). The objective is to determine whether the patient requires intervention, defined as transfusion, endoscopic or surgical intervention, death, or rebleeding (Blatchford et al., 2000). According to the score, patients can be divided into medium-high risk (≥6 scores) and low-risk (<6 scores). Specific scores are shown in Table 2.

**Table 2:** Blatchford ratings.

Variables	Score
Heart rate (beats per minute)	
≥100	1
Heart rate (beats per minute)	
100–199	1
90–99	2
<90	3
Heart rate (beats per minute)	
6.5–7.9	2
8.0–9.9	3
10.0–24.9	4
>25.0	6
Hemoglobin (mmol/L)	
Male 120–130	1
110–119	3
<100	6
Female 100–120	1
<100	6
Other indicators	
Pulse ≥ 100	1
Accompanied by black stool	1
Present as syncope	2
Liver disease	2
Heart failure	2

#### 2.2.3 AIMS65 score

The AIMS65 score, a preendoscopic risk score published in 2011, included 29,222 UIGB patients admitted to 187 hospitals from 2004 to 2005. AIMS65 was externally validated 1 year later, and a total of 32,504 patients were included in the database for its development. On a scale of 0 to 5, AIMS65 includes five clinical or laboratory variables: age of onset; Albumin, INR (as an indicator of coagulation function of patients, “INR” is an abbreviation of the international standardized ratio, derived from the international sensitivity index of prothrombin time and determination reagents); Changes in mental status and systolic blood pressure. Used to predict patient mortality (Saltzman et al., 2011). According to the score, patients can be classified into high risk (≥2 scores) and low risk (<2 scores). Specific scores are shown in Table 3.

**Table 3:** AIMS65 score content.

Variables	Score
Albumin < 30 g/L	1
INR > 1.5	1
Altered mental state	1
Systolic blood pressure (mmHg) ≤ 90	1
Age > 65 years	1

Three scores were used to perform risk scores for the enrolled patients and compared with final patient outcomes and predictions in the deep learning model to show the efficacy of the current scoring model.

### 2.3 Methods for determining rebleeding

According to the clinical manifestations of the patient during the hospital, such as whether the patient has hematemesis, hematochezia or black stool, and the changes of the patient’s clinical indicators, such as changes in the hemoglobin level and coagulation function such as platelets during the hospital stay, if the patient has undergone endoscopy during the hospital stay, a comprehensive judgment will be made on whether the patient has rebleeding during the hospital stay combined with the contents of the endoscope.

### 2.4 Machine learning methods

Four machine learning methods were used in this study, including Decision Tree (DT), Random Forest (RF), Logistic Regression (LR), K-Nearest Neighbors (KNN).

DT is a classification or regression model with a tree-like structure that splits a sample into different branches by conditions to ultimately generate a prediction result. Maximize the information gain (or reduce the Gini coefficient) after each partition by dividing the input feature space. The gradient of the tree is generated from the root node to the leaf node, and the leaf node stores the prediction results. It is fast to train, easy to visualize, does not require data consumption (such as normalization), can handle multiple classification problems, but is sensitive to data noise. RF is an integrated learning algorithm based on decision trees that builds multiple decision trees and outputs the results by voting. The bagged method is used to randomly sample multiple sub-data sets from the original data. Each tree selects some features during training and obtains the final prediction result by voting (classification) or averaging (regression). It is suitable for classification and regression, and can handle nonlinear relationships between high-dimensional data and features. But computation costs are high, training and prediction are slow, and interpretative differences are output. LR is a linear model used for binary classification problems. It predicts the probability of an event occurring by using a linear combination of feature inputs and converts the output to a probability value between 0 and 1 using the Sigmoid function. The model is simple, easy to understand and implement, and can provide a probability value for each predicted output to explain and make decisions. At the same time, compared with other complex models, it has faster training and prediction speed and wider applicability, but it is more sensitive to noise and outliers, which may affect the model performance and can only handle binary classification problems. KNN algorithm is an instance-based learning method used for classification and regression problems. It does not go through an explicit training process, but instead calculates the distance (usually using Euclidean distance or Mahalanobis distance) between the predicted sample and the training set samples directly, and finds the nearest K neighbors. The prediction is made based on the categories (or numerical values) of these neighbors. Its transformation is simple to implement and easy to understand, and does not require a training process. It is suitable for problems without explicit distribution assumptions and can handle classification and regression problems, as well as multi-class classification. However, its computational cost is high and it is not suitable for high-dimensional data with irregularities.

### 2.5 Deep learning model

This study classifies the information into four major categories: clinical information (including gender, age, concomitant diseases such as heart failure, ischemic heart disease, and other significant concomitant diseases, liver failure, renal failure, cancer metastasis, other manifestations, and diabetes history), vital signs (including systolic blood pressure, syncope, heart failure), blood indicators (including the degree of infection, hemoglobin, INR, and blood urea nitrogen), and stool examination (melena).

This study proposes a novel model based on Transformer and Kolmogorov–Arnold Networks (KAN), where the final linear layer of the Transformer is replaced by KAN. In the structure of the model, after each category of information is processed by the Transformer, one feature value is output. Finally, the feature values of the four types of information are concatenated and input into the final classification layer (KAN). This fusion structure not only combines the powerful feature extraction capability of Transformer, but also generates symbolic formula by introducing KAN to facilitate the analysis of the influence of different dimension information on the prediction results. The performance evaluation of the model is conducted by plotting the line graphs of the loss function (loss) and accuracy (accuracy). This approach visually demonstrates the convergence of the model during the training and validation processes as well as the changing trend of the classification performance, facilitating the assessment of the model’s stability and generalization ability. In order to evaluate the generalization ability of deep learning models more comprehensively and stably, and make full use of limited data to avoid overfitting, this paper used five-fold cross validation to test the model.

In addition, we wanted to explore whether the infection situation would affect the prediction efficiency of the deep learning model. We designed two comparative experiments: one was to predict the best trained model in the *H. pylori* infected group and the *H. pylori* non-infected group; the other was to evaluate the prediction efficiency of the best model by setting the degree of infection as irrelevant random noise.

### 2.6 Statistics methods

IBM SPSS 26.0 software was used for statistical analysis. We first run the Kolmogorov–Smirnov normality test on all the data. For binary classification, the Mann–Whitney U test was used for inter-group differences of continuous variables, Chi-square test was used for inter-group differences of class variables, and Kruskal-Wallis H test was used for multi-class variables and continuous variables. The odds ratio and 95% confidence interval were increased for Kruskal-Wallis H test and Chi-square test. For Mann–Whitney U test, r effect size was increased. *p* < 0.05 was considered statistically significant.

**Figure 2:**
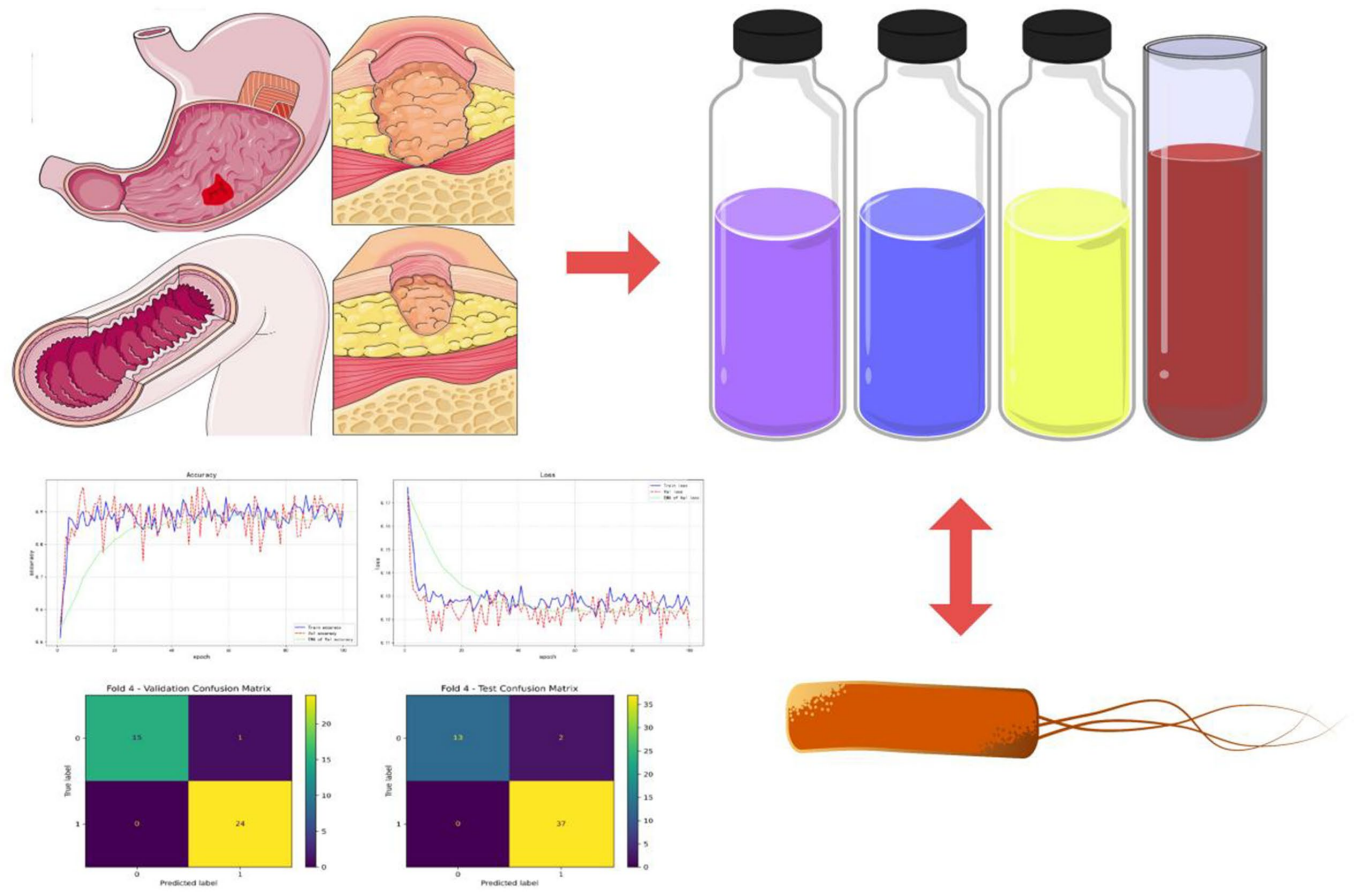
Experimental flow chart. Various laboratory indicators such as blood tests and stool tests were gathered from patients with acute upper gastrointestinal bleeding. In combination with the patients’ *Helicobacter pylori* infection, a risk prediction model for rebleeding in inpatients with UGIB was constructed.

The flow chart of the experiment is shown in Figure 2.

## 3 Results

### 3.1 Baseline

A total of 254 patients were included in the study, and variables with missing values greater than 20% were excluded. Table 4 shows clinical information and laboratory parameters for included patients.

**Table 4:** Basic information of patients.

	Rehaemorrhagia	Normal
Sex	M130/F20	M78/F26
Age	52.23 (49.44,55.01)	53.85 (50.67,57.02)
Concomitant diseases	Y32/N118	Y21/N83
Whether have diabetes	Y15/N135	Y11/N93
Systolic pressure	121.01 (117.96,124.06)	123.28 (119.73,126.82)
Whether syncope	Y1/149	Y7/97
Whether heart failure	Y0/N150	Y0/N104
Degree of infection	N60/A9/B1/C22/D58	N29/A8/B3/C20/D44
Hemoglobin	92.08 (88.13,96.03)	93.32 (87.73,98.91)
INR	0.89 (0.77,1.01)	0.98 (0.77,1.20)
Blood urea nitrogen	10.72 (2.51,18.92)	6.32 (5.37,7.27)
Ulcer lesions	Y87/N63	Y56/N48
Tarry stool	Y131/N19	Y89/N15

### 3.2 Correlation between rebleeding and various indicators

#### 3.2.1 Correlation between rebleeding and clinical information

Table 5 shows the correlation between rebleeding in included patients and clinical information. In the correlation analysis between rebleeding and clinical data, the risk of rebleeding in males (62.5%) was higher than that in females (43.47%) (*p* < 0.05, with statistical significance). The risk of rebleeding increased with age (*p* > 0.05, without statistical significance). The risk of rebleeding in patients with combined disease (60.37%) was higher than that in patients without combined disease (58.70%) (*p* < 0.05, with statistical significance). The risk of rebleeding was higher in patients without diabetes (59.21%) than in patients with diabetes (56.00%) (*p* < 0.05, without statistical significance).

**Table 5:** Correlation between rebleeding and clinical information.

	Statistic	*p* value	Statistical method	r effect size	Odds ratio	95%CI lower	95% CI upper
Sex	4.8776	0.0272^*^	Chi-square test		2.1667	1.1344	4.1380
Age	8449.5	0.2595	Mann–Whitney U test	0.0708			
Concomitant diseases	0.0040	0.9497	Chi-square test		1.0719	0.5778	1.9883
Whether have diabetes	0.7921	0.6730	Chi-square test		0.8768	0.3813	2.0162

^*^The difference is statistically significant (*p* < 0.05).

#### 3.2.2 Correlation between rebleeding and vital signs

Table 6 shows the correlation between rebleeding and vital signs in included patients. In the correlation analysis between rebleeding and vital signs, the risk of rebleeding gradually increased with the increase of systolic pressure (*p* > 0.05, without statistical significance), and the risk of rebleeding in patients without fainting (60.57%) was higher than that in patients with fainting (12.50%) (*p* < 0.05, with statistical significance).

**Table 6:** Correlation between rebleeding and vital signs.

	Statistic	*p* value	Statistical method	r effect size	Odds ratio	95%CI lower	95% CI upper
Systolic pressure	8458.5	0.2510	Mann–Whitney U test	0.0720			
Whether fainting	5.5495	0.0185^*^	Chi-square test		0.0930	0.0113	0.7678

^*^The difference is statistically significant (*p* < 0.05).

#### 3.2.3 Correlation between rebleeding and laboratory examination items

Table 7 shows the correlation between rebleeding in included patients and the items examined in the laboratory. In the correlation analysis between rebleeding and laboratory examination items, the probability of rebleeding did not change with the increase of *H. pylori* infection degree (*p* > 0.05, without statistical significance). With the changes of hemoglobin, INR and blood urea nitrogen, the probability of rebleeding would also change (*p* > 0.05, without statistical significance).

**Table 7:** Correlation between rebleeding and laboratory examination items.

	Statistic	*p* value	Statistical method	r effect size	Odds ratio	95%CI lower	95% CI upper
Degree of infection	5.7306	0.2202	Chi-square test		0.5438	0.1902	1.5546
Hemoglobin	7916.5	0.8403	Mann–Whitney U test	0.0126			
INR	7,787	0.9822	Mann–Whitney U test	0.0014			
Blood urea nitrogen	7821.5	0.9709	Mann–Whitney U test	0.0023			

#### 3.2.4 Correlation between rebleeding and stool examination

Table 8 shows the correlation between rebleeding and stool examination in included patients. In the correlation analysis between rebleeding and stool examination, the risk of rebleeding in patients with black stool (59.55%) was higher than that in patients without black stool (55.88%) (*p* > 0.05, without statistical significance).

**Table 8:** Correlation between rebleeding and stool examination.

	Statistic	*p* value	Statistical method	Odds ratio	95%CI lower	95% CI upper
Tarry stool	0.0470	0.8283	Chi-square test	1.1620	0.5608	2.4078

#### 3.2.5 Correlation between *Helicobacter pylori* infection degree and laboratory examination items

Table 9 shows the correlation between *H. pylori* infection degree and laboratory examination items of included patients. In the correlation analysis between the degree of *H. pylori* infection and laboratory examination items, the hemoglobin level of patients changed with the degree of infection (*p* < 0.05, with statistical significance), and the other laboratory indicators of patients also changed with the degree of infection (*p* > 0.05, without statistical significance).

**Table 9:** Correlation between degree of infection and stool examination.

	Statistic	*p* value	Statistical method	95%CI lower	95% CI upper
Hemoglobin	11.6905	0.0198^*^	Kruskal-Wallis H test	4.6882	30.3764
INR	1.8235	0.7682	Kruskal-Wallis H test	0.8621	14.6232
Blood urea nitrogen	2.3471	0.6722	Kruskal-Wallis H test	0.8160	15.7447

^*^The difference is statistically significant (*p* < 0.05).

### 3.3 Comparison between three common ratings, machine learning algorithm and deep learning algorithm model

#### 3.3.1 Results of three common ratings

The three scores for the patients in this study are shown in Table 10.

**Table 10:** Three categories of ratings and their detection rates.

	RS	GBS	AIMS65 score
Low risk	213	58	254
Middle and high risk	41	196	0
Detection rate	16.14%	77.17%	0

#### 3.3.2 Comparison of accuracy of four machine learning scoring methods

**Figure 3:**
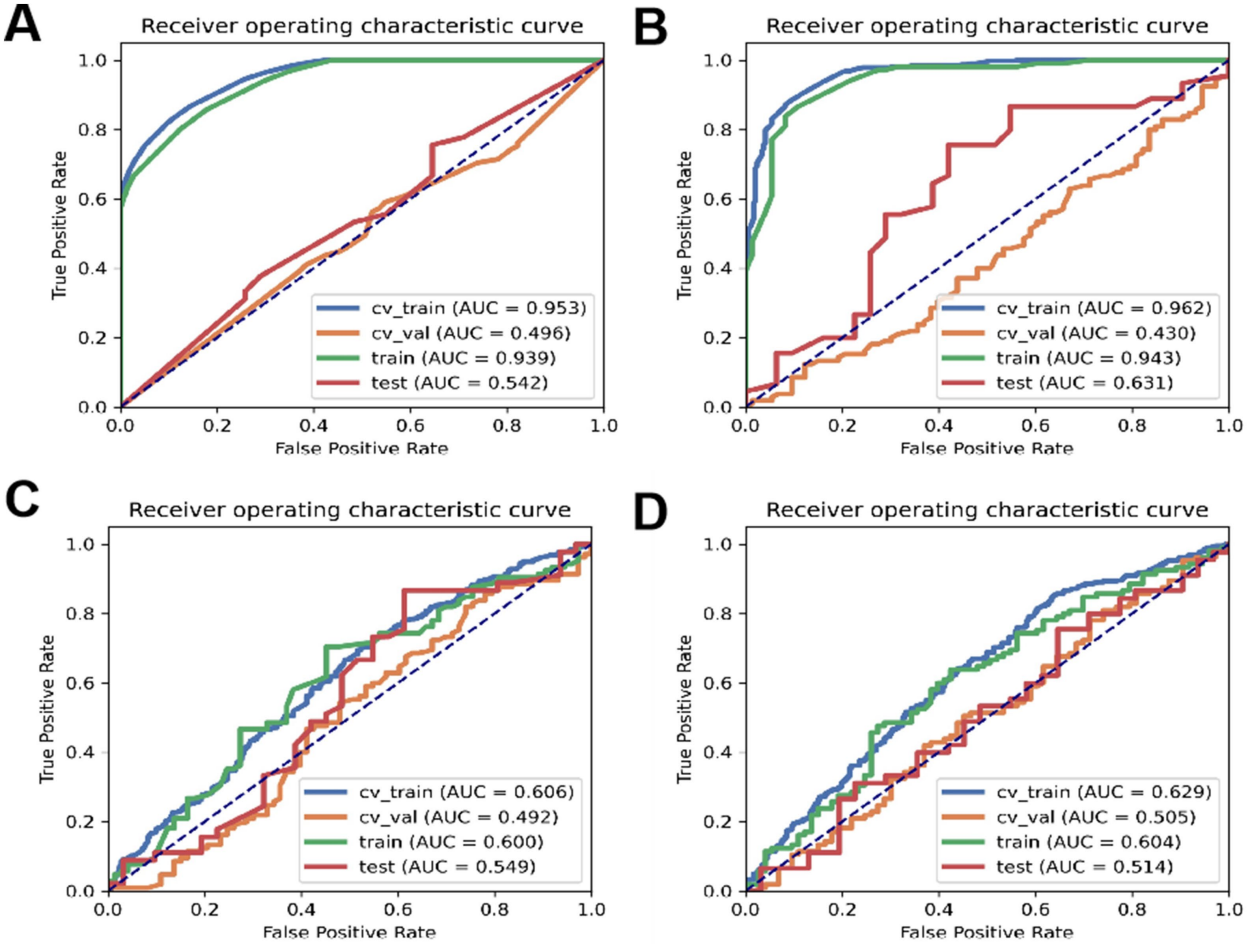
**(A)** ROC curve of DT, **(B)** ROC curve of RF, **(C)** ROC curve of LR, **(D)** ROC curve of KNN.

According to Table 11, the accuracy of the test set for DT analysis of machine learning methods is 0.53; the accuracy of the test set of RF machine learning method is 0.68; the accuracy of the test set of the machine learning method of LR machine learning method is 0.67; the accuracy of the test set of KNN machine learning method is 0.59. ROC curves for the four types of machine learning are shown in Figure 3.

**Table 11:** Results of machine learning algorithm.

	Accuracy	Sensitivity	Specificity	PPV	NPV	AUC	95%CI Lower	95% CI Upper
DT	0.5337	0.5905	0.4521	0.6078	0.4342	0.4959	0.4145	0.5808
RF	0.6842	0.7556	0.5806	0.7234	0.6207	0.6312	0.4933	0.7667
LR	0.6711	0.8667	0.3871	0.6724	0.6667	0.5491	0.4073	0.6889
KNN	0.5921	0.7556	0.3548	0.6296	0.5	0.514	0.3777	0.6449

#### 3.3.3 Effect of deep learning model

**Figure 4:**
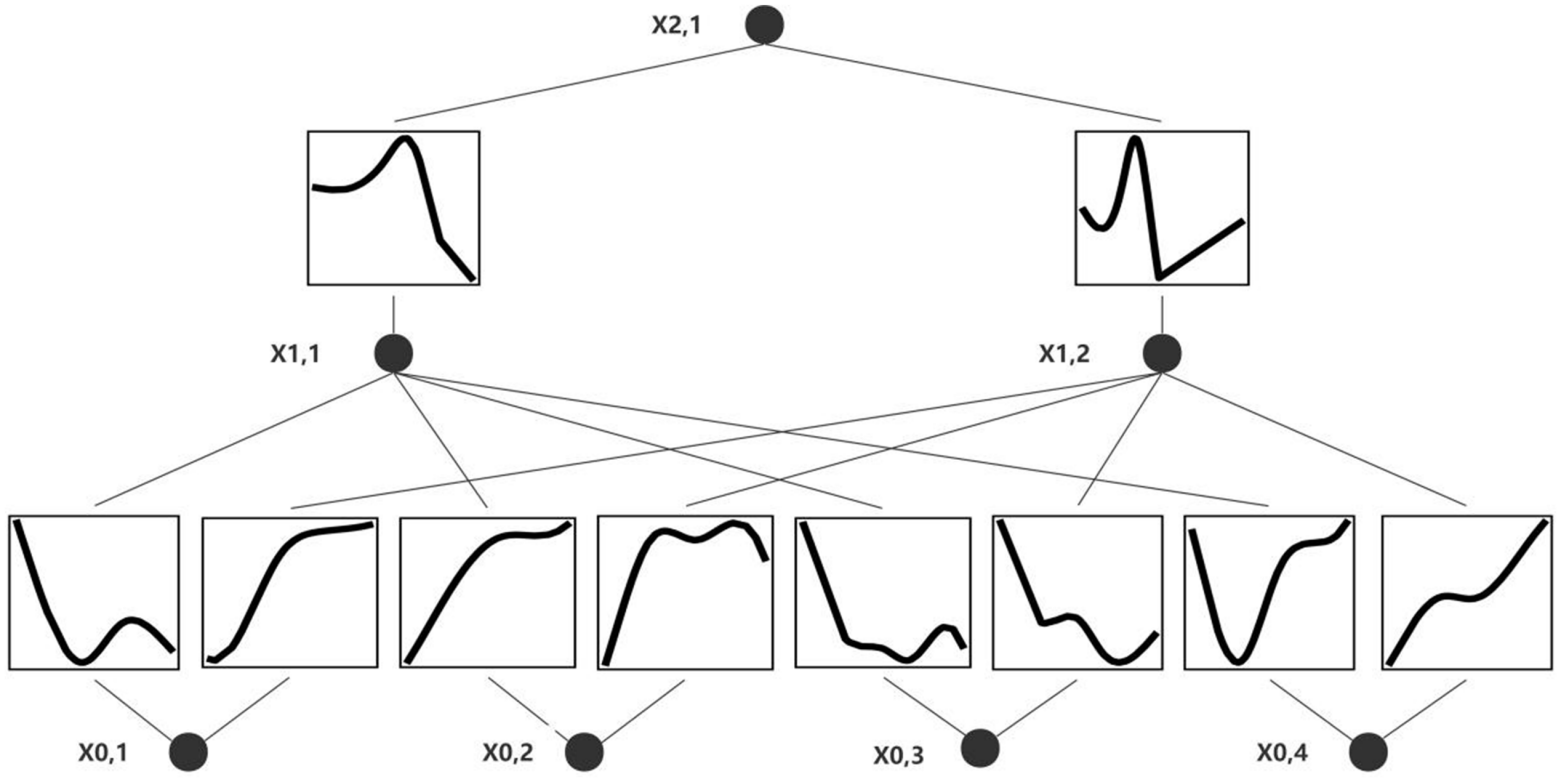
Deep learning model visualization. x0,1: clinical information, x0,2: vital signs, x0,3: blood parameters, x0,4: stool examination.

In this study, after processing each type of information by transformer, a characteristic value will be output, and the characteristic value will be represented by x1-4, respectively. x1-4 represents clinical information, vital signs, laboratory examination items and stool examination respectively, which can be understood as dimensionally reduced by transformer. Finally, x1-4 is spliced and input into the final classification layer (KAN) to make predictions and provide symbolic formula: 0.32*x_1 + 1.61*x_2 – 0.42*x_3 + 0.23*x_4 – 0.23* tanh (8.13*x_1 + 1.14) + 0.54. The formula combines linear terms and hyperbolic tangent functions to capture the multilevel and nonlinear effects of the input variables (x_1, x_2, x_3, x_4) on the target variable (y). The formula can express complex variable relationships and reveal the mode of action of different factors in the risk of recurrent gastrointestinal bleeding. The flow chart of the KAN model is shown in Figure 4.

**Figure 5:**
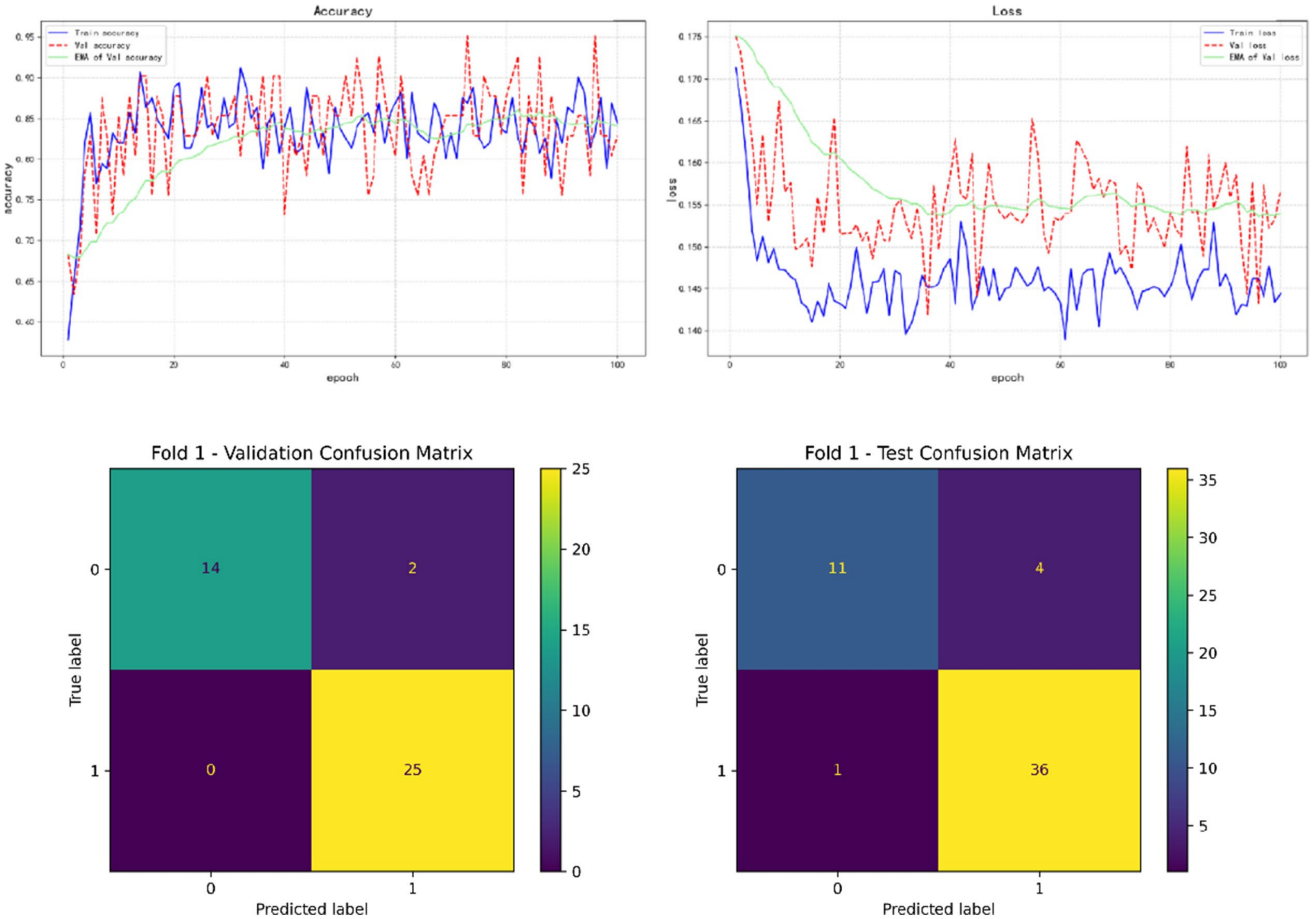
Fold 1 results and Validation confusion matrix and Test confusion matrix.

**Figure 6:**
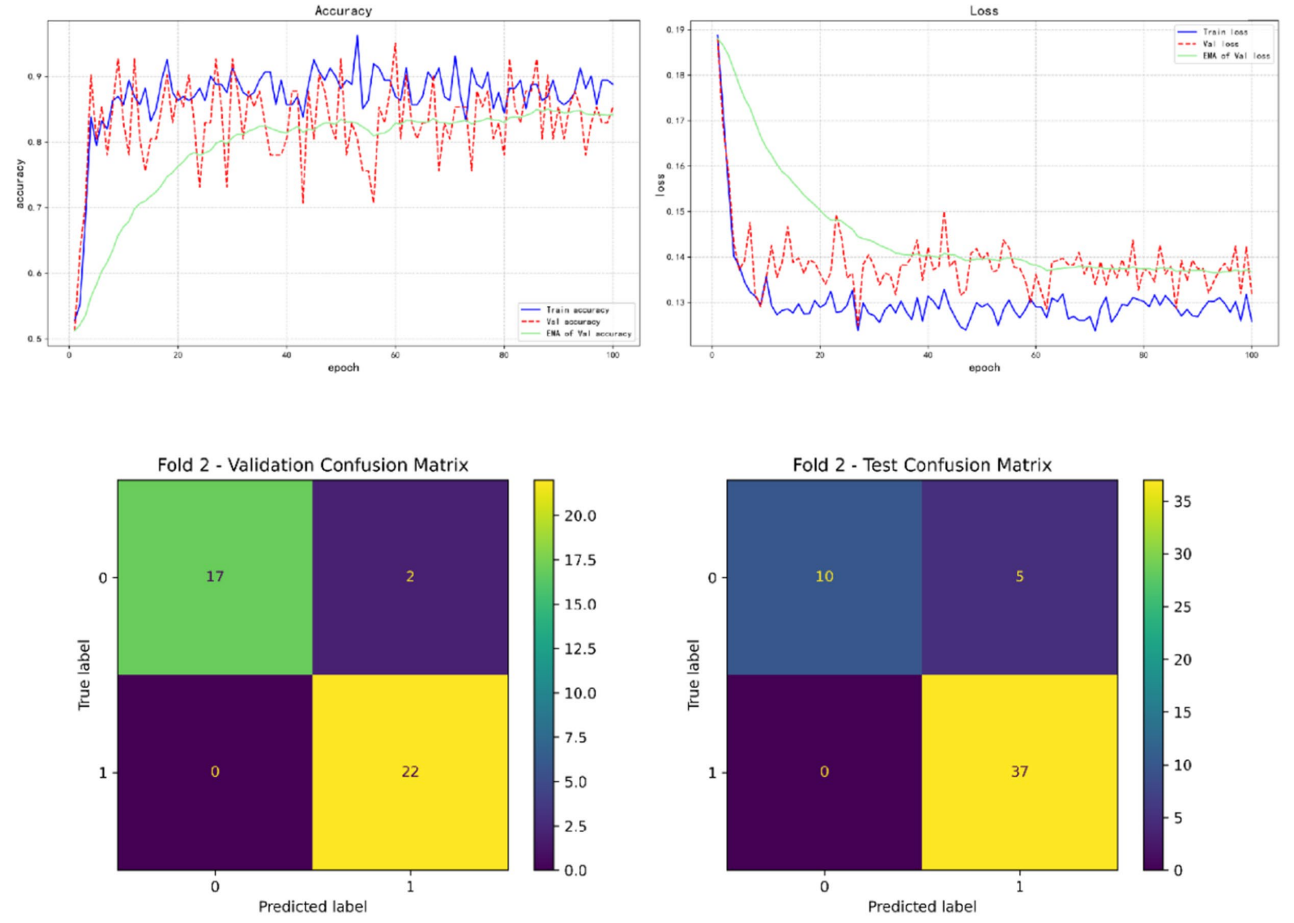
Fold 2 results and validation confusion matrix and test confusion matrix.

**Figure 7:**
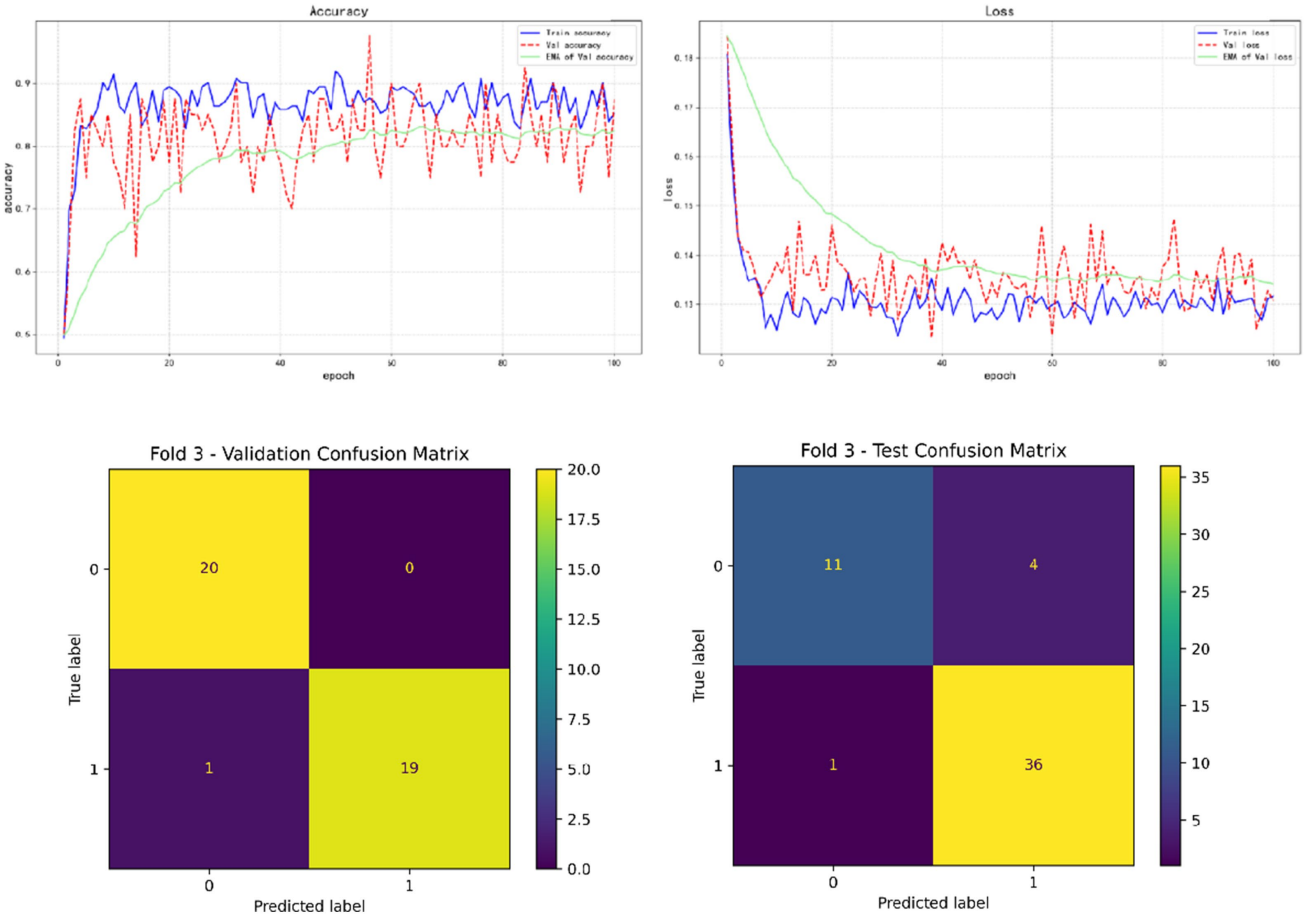
Fold 3 results and validation confusion matrix and test confusion matrix.

**Figure 8:**
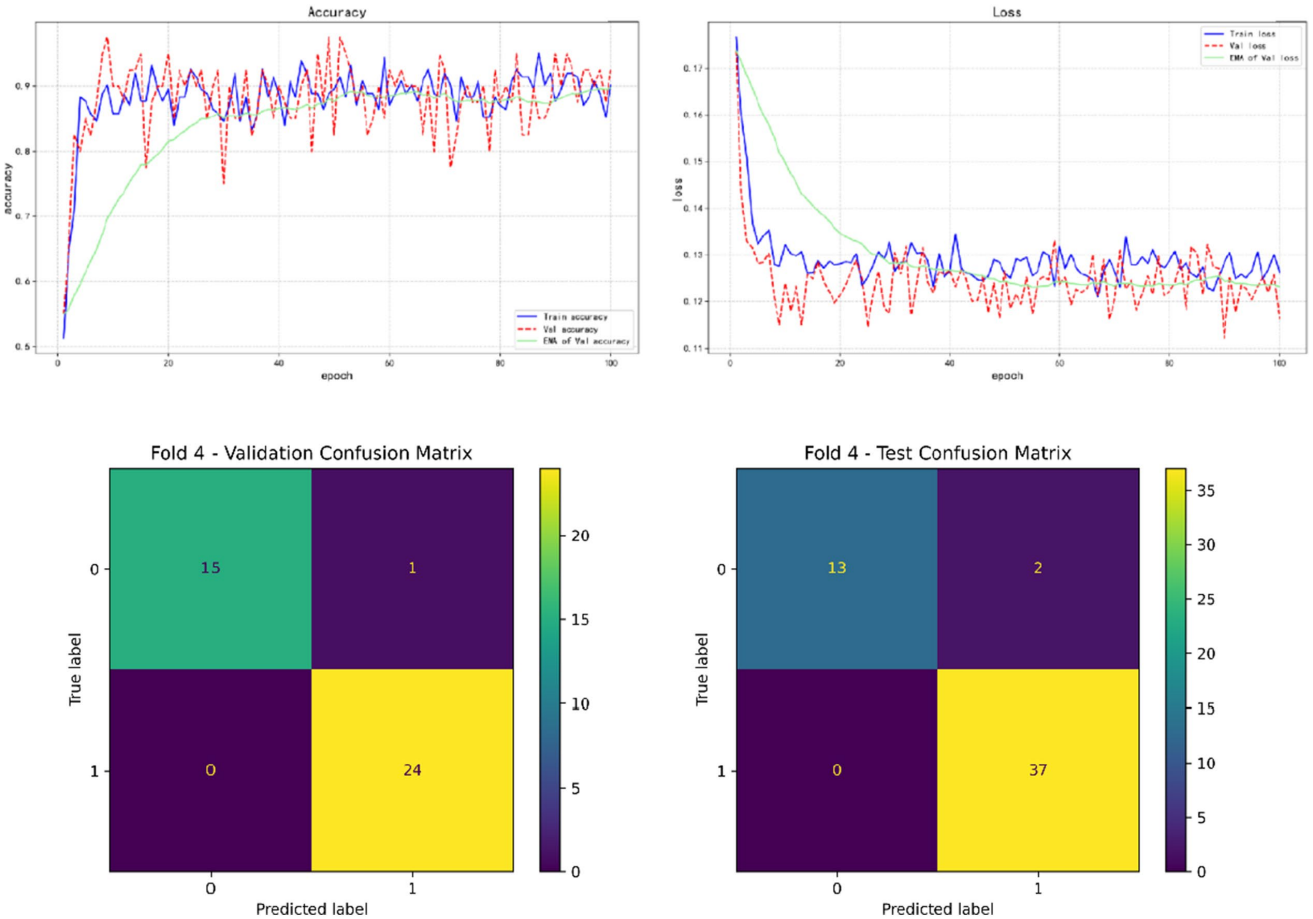
Fold 4 results and validation confusion matrix and test confusion matrix.

**Figure 9:**
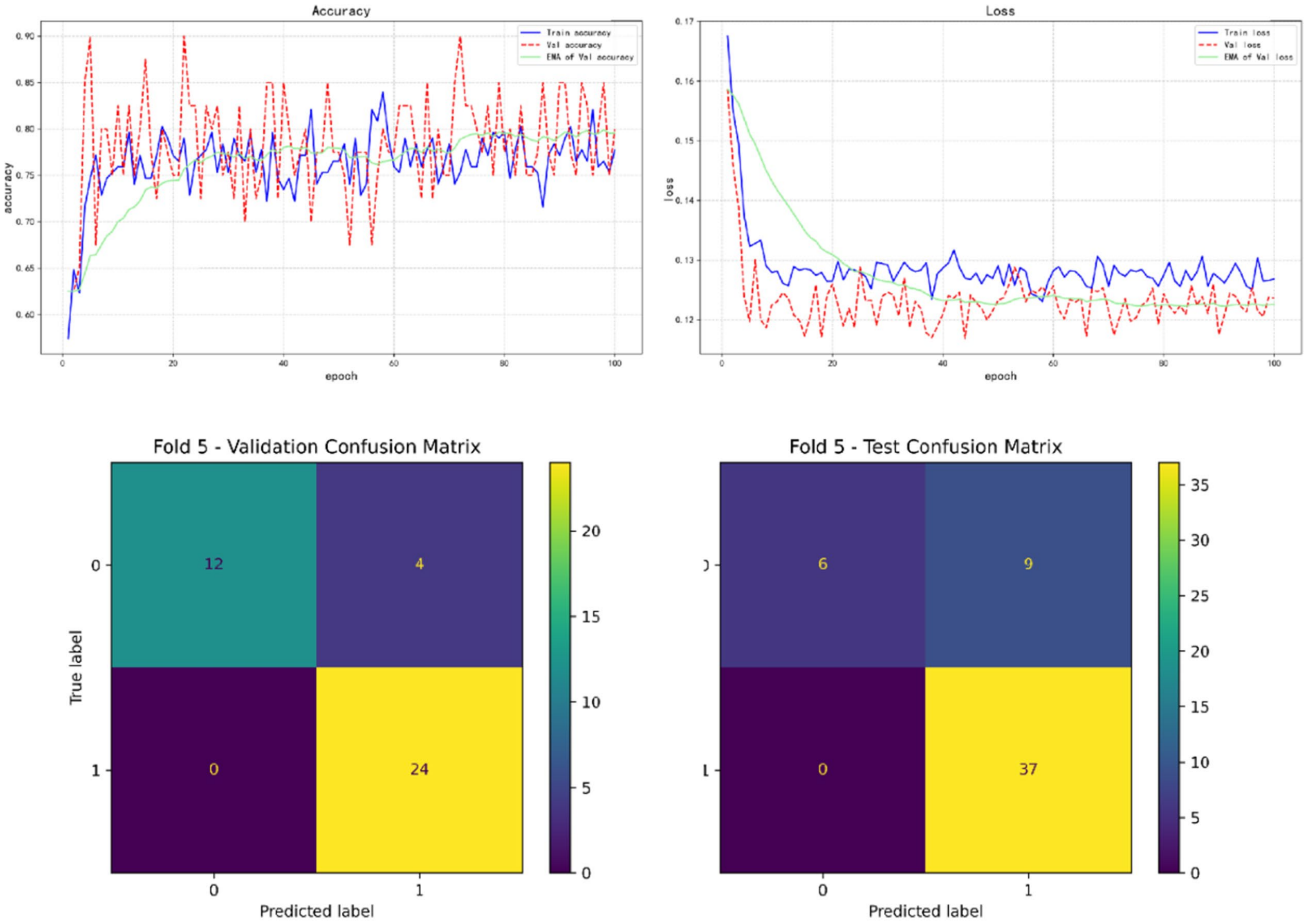
Fold 5 results and validation confusion matrix and test confusion matrix.

In the results of five-fold cross validation, the training performance of the fourth fold is particularly prominent, and the model achieves the best prediction accuracy on both the validation set (accuracy = 0.9750) and the test set (accuracy = 0.9615). In addition, by comparing the accuracy and loss curves in the training process, better fitting effect and more stable gradient descent trend can be observed, which further verifies the robustness of the training. The results of the five-fold cross validation and the results of the confusion matrix are shown in Figures 5–9 and Table 12.

**Table 12:** Result of five-fold cross validation.

		Accuracy	Precision	Recall	F1-score
Fold 1	Validation set	0.9512	0.9259	1.0000	0.9615
	Test set	0.9038	0.9000	0.9730	0.9351
Fold 2	Validation set	0.9512	0.9167	1.0000	0.9565
	Test set	0.9038	0.8810	1.0000	0.9367
Fold 3	Validation set	0.9750	1.0000	0.9500	0.9744
	Test set	0.9038	0.9000	0.9730	0.9351
Fold 4	Validation set	0.9750	0.9600	1.0000	0.9796
	Test set	0.9615	0.9487	1.0000	0.9737
Fold 5	Validation set	0.9000	0.8571	1.0000	0.9231
	Test set	0.8269	0.8043	1.0000	0.8916

In addition, by exploring the influence of infection on the model prediction, it was found that the prediction accuracy of rebleeding in the non-*H. pylori* infection group (0.8989) was lower than that in the *H. pylori* infection group (0.9636), and other evaluation parameters were also lower than that in the infection group. In addition, by adding irrelevant random noise to mask the influence of infection degree on model output, it is found that the model prediction accuracy (0.7992) is significantly reduced. The results of the comparison experiment are shown in Table 13.

**Table 13:** The results of the comparison experiments.

	Accuracy	Precision	Recall	F1-score
Non-infection group	0.8989	0.9180	0.9333	0.9256
Infection group	0.9636	0.9565	0.9778	0.9670
Noised infection level group	0.7992	0.7765	0.9267	0.8450

## 4 Discussion

Based on four types of data (including vital signs, clinical information, blood indicators and stool detection) of UGIB patients infected with *Helicobacter pylori* (*H. pylori*), this study uses Transformer network as a feature extractor and KAN network as a classifier to build a deep learning prediction model. To predict the risk of rebleeding in UGIB patients during hospitalization, and compared with four machine learning models, good results were obtained, and the accuracy of the final model reached 0.9615.

In a model that predicted the risk of gastrointestinal bleeding rebleeding, the effect of vital sign depth features was positive. Tari et al. (2023) found that UGIB patients with impaired hemodynamics were at increased risk for all associated adverse outcomes, such as higher rates of hospitalization and increased rates of re-bleeding within 30 days. In addition, UGIB patients with impaired hemodynamics require surgery more frequently. This result is consistent with the conclusion of the model in this study that abnormal vital signs are associated with an increased risk of rebleeding.

Secondly, although the clinical information depth feature presents a positive linear relationship, due to the hyperbolic tangent (tanh) nonlinear term contained in it, the information may lead to complex fluctuations under different circumstances. The depth characteristics of blood indicators showed a relatively small negative relationship. In most studies on the risk of UGIB rebleeding, different models analyzed different clinical information and blood markers and achieved better results, although the specific information and markers were different (Ungureanu et al., 2023; Uysal, 2021; Zhuang et al., 2023). However, in a recent study by Taylor et al. (2025), blood type was found to be associated with the risk of upper gastrointestinal bleeding, thrombosis and peptic ulcer disease. Blood type B is associated with a reduced risk of overall outcomes, including rebleeding, the need for surgery or embolization, and mortality, compared to non-B blood type. In part, this may explain that even when the same blood indicators or clinical information is used, their contribution to the prediction of rebleeding in patients may vary. Finally, fecal depth features showed a slight positive effect on the risk of recurrence, suggesting that fecal occult blood suggests the risk of rebleeding. Compared to deep learning models, the lower prediction accuracy of traditional machine learning models is due to their limited ability to handle highly nonlinear data or complex relationships, making it difficult to capture the intrinsic connections between various indicators. Deep learning, on the other hand, overcomes this limitation by seamlessly linking the internal relationships among all indicators, integrating them into a unified whole to accomplish tasks.

In the correlation between the risk of rebleeding and various indicators, we found that gender was related to the risk of rebleeding, that is, men were more likely to have rebleeding during hospitalization for upper gastrointestinal bleeding, which was also demonstrated in the study of Jeon et al. (2021), which found that gender could be used as a set of variables to predict rebleeding in UGIB patients. Similarly, the study results of Snipe et al. show that when women are in the follicular phase of the menstrual cycle, biological sex has no effect on intestinal epithelial injury and permeability, and has the least effect on gastrointestinal symptoms and the systemic cytokine spectrum in response to stress of exhaustion. However, the influence of males after exercise is greater than that of females (Snipe and Costa, 2018). Therefore, whether to keep calm after detecting the symptoms of upper gastrointestinal bleeding? Avoiding unnecessary exercise can reduce the risk of rebleeding, which is worth exploring. And in the study by Zheng et al. (2022), it can be seen that although the incidence of UGIB has decreased in recent years, the age/sex-adjusted incidence of GIB increased from 378.4 per 100,000 people to 397.5 per 100,000 people between 2006 and 2019, so it is important to focus on the elderly. In particular, the incidence of UGIB in older men and the risk of rebleeding after hospitalization are necessary and meaningful behaviors. In the scoring system studied by Jeon et al. (2021), whether syncope is associated with mortality within 30 days after hospitalization in UGIB patients, syncope and rebleeding risk obtained in this experiment can supplement this experiment to some extent. Heyer et al. (2018) found that nausea associated with syncope was related to the change of gastric myoelectric activity and the increase of vasopressin and epinephrine in time, which could also explain that syncope patients were more likely to induce rebleeding to a certain extent, and the related mechanism of syncope and rebleeding could be further explored in future studies. In addition, in two reports on rare cases of upper gastrointestinal bleeding (Li et al., 2024; Ulbricht et al., 2022), syncope occurred in both patients. Although the internal correlation between syncope and rebleeding has not been actually explored, it can also suggest that current UGIB patients should pay attention to the clinical changes if syncope occurs. To provide clinical improvement measures as soon as possible. In the new UGIB treatment management, for all UGIB patients, it is recommended to resuscitate with intravenous infusion and red blood cell infusion according to the need, and the hemoglobin threshold is 70–80 g/L. When the hemoglobin of UGIB patients is lower than this threshold, resuscitation and red blood cell infusion therapy are performed (Stanley and Laine, 2019), which shows the influence of hemoglobin value on clinical treatment decision making. The new treatment management is based on the patient’s current vital signs and does not investigate how the remaining risks during hospitalization are reflected in the hemoglobin value, however, a number of studies have been involved in the study of hemoglobin and the risk of rebleeding, and it is found that there is a certain correlation between hemoglobin and rebleeding. In addition, Extrat et al.’s study, hemoglobin level after arterial embolization in UGIB patients is more likely to reflect the early mortality and the risk of rebleeding in patients (Extrat et al., 2022; Tatlıparmak et al., 2022; Zheng et al., 2019). This has further defined the research direction for clinical research, which can be focused on in future studies. In addition, there is still some controversy about the hemoglobin threshold for red blood cell transfusion (Carson et al., 2021; Kola et al., 2021; Laine et al., 2021; Page et al., 2021; Teutsch et al., 2023), and the results of individual studies show that if the transfusion is started with a lower threshold, the incidence of transfusion reaction and post-transfusion intervention is lower. A hemoglobin threshold greater than 80 g/L may result in a higher rate of adverse outcomes. In conclusion, the correlation mechanism between hemoglobin value and clinical events during hospitalization should be explored, and then the optimal restrictive transfusion threshold should be further studied. As the most direct sign of UGIB diagnosis, endoscopy is usually the most concerned result in clinic. However, limited by the scale and the use of instruments in some small and medium-sized hospitals, the current research focuses on the diagnosis of UGIB by skipping endoscopy or replacing endoscopy or predicting clinical outcome.

In addition, we wanted to explore whether the infection situation would affect the prediction efficiency of the deep learning model. We designed two comparative experiments: one was to predict the best trained model in the *H. pylori* infected group and the *H. pylori* non-infected group; the other was to evaluate the prediction efficiency of the best model by setting the degree of infection as irrelevant random noise. Although the correlation analysis did not find significant correlation between the degree of *H. pylori* infection and the risk of rebleeding. This may be due to the uneven distribution of data on the degree of certain infections or the fact that the infection affects the risk of rebleeding in a more complex non-linear relationship. However, compared with non-infected patients, it can be found from the results of the comparative experiment that the model has a higher prediction accuracy for rebleeding risk in infected patients with subdivided infection degree. Meanwhile, when the information of infection degree is ignored in the model, the prediction efficiency of the model is greatly reduced. Therefore, we believe that *H. pylori* infection plays a crucial role in the risk prediction of rebleeding, and *H. pylori* infection may affect the final prediction results in a non-linear manner by influencing other factors or complex combinations of variables. Although few studies have included the degree of *H. pylori* infection in the diagnosis and treatment of upper gastrointestinal bleeding (UGIB), there have been studies that have analyzed the correlation between the diagnosis of *H. pylori* infection and gastroscopy (EGD) results in other gastrointestinal diseases, such as gastric and duodenal ulcers. Attempts were made to replace gastroscopy to some extent by non-invasive detection of *H. pylori* infection (Liao et al., 2023). In the Pritchard DM study, the use of *H. pylori* infection testing as an alternative to gastroscopy and treatment was found to be the most cost-effective strategy (Pritchard et al., 2021). At the same time, *H. pylori* diagnostic methods have been increasingly improved, such as serological detection, fecal antigen detection and urea breath test, all of which are practical and highly sensitive (Ghazanfar et al., 2024). This study also explored the value of incorporating *H. pylori* infection in predicting the risk of rebleeding in patients with upper gastrointestinal hemorrhage during hospitalization.

At present, for the risk of rebleeding during hospitalization in UGIB patients, in addition to timely and accurate endoscopy, there are multiple scoring mechanisms. We found that the three types of traditional scoring are for different emergency time periods, and there are some problems in the detection efficiency, and the scoring also represents the risk in different time periods, and there is a certain lag or inaccuracy in clinical measures, but the four types of machine learning scoring methods can integrate all factors and examinations to evaluate the overall period of hospitalization of patients. Patients were monitored as a whole, but the accuracy of machine learning is not satisfactory. Meanwhile, deep learning models with higher testing efficiency provide stronger technical support for overall detection. For patients with UGIB, collecting relevant information based on deep learning models helps to make the fastest and most accurate judgments in the absence of endoscopy. However, this study is limited to a single center and lacks validation from multicenter experimental data, which will be an important direction for future research.

## 5 Conclusion

Based on the degree of *H. pylori* infection in patients with upper gastrointestinal bleeding, combined with a number of clinical laboratory tests and clinical data, we developed a clinical model for predicting the risk of rebleeding in patients with upper gastrointestinal bleeding. It provides an early prediction of rebleeding during a patient’s hospitalization and optimizes early intervention for patients to a certain extent. It provides a more concise, convenient and effective guidance scheme for small and medium-sized hospitals to make clinical decisions for UGIB patients.

## Data Availability

The raw data supporting the conclusions of this article will be made available by the authors, without undue reservation.
